# Effect of Curcumin Supplement in Summer Diet on Blood Metabolites, Antioxidant Status, Immune Response, and Testicular Gene Expression in Hu Sheep

**DOI:** 10.3390/ani9100720

**Published:** 2019-09-24

**Authors:** Zhiyang Jiang, Yongjie Wan, Peng Li, Yang Xue, Wenwen Cui, Qi Chen, Jianqin Chen, Feng Wang, Dagan Mao

**Affiliations:** 1 College of Animal Science and Technology, Nanjing Agricultural University, Nanjing 210095, Jiangsu, China; 2018805102@njau.edu.cn (Z.J.); wanyongjie@njau.edu.cn (Y.W.); 2018105018@njau.edu.cn (Y.X.); cuiwenwen0602@163.com (W.C.); clinqda@gmail.com (Q.C.); 2018805105@njau.edu.cn (J.C.); caeet@njau.edu.cn (F.W.); 2 National Experimental Teaching Demonstration Center of Animal Science, Nanjing Agricultural University, Nanjing 210095, Jiangsu, China; 3 Qidong Ruipeng Animal Husbandry Co., Ltd, Nantong 226227, Jiangsu, China; lipeng1411@163.com

**Keywords:** curcumin, Hu sheep, high temperature environment, blood biochemical parameters, testis, gene expression

## Abstract

**Simple Summary:**

Heat stress can induce oxidative stress and has an adverse effect on the growth and reproductive performance in animals. Curcumin, a plant-derived substance, with the effect of scavenging oxidative free radicals, improving immune response and anti-apoptosis, has been widely used as a dietary supplement in the livestock industry. The present study aims to investigate the effect of a curcumin dietary supplement on the blood metabolites, antioxidant status, immune response, and testicular gene expression in Hu Sheep in summer. The results show that dietary curcumin supplementation (450 and 900 mg/per sheep daily) can promote lipid metabolism, antioxidant capacity, and immune response as well as testicular development in Hu sheep, which provides evidence for the protective role of curcumin against heat stress in sheep.

**Abstract:**

In summer, the high temperature affects animal growth and reproductive performance. Curcumin is a flavonoid with anti-oxidant and anti-inflammatory effects. To evaluate the effects of dietary curcumin supplement on the blood biochemical parameters and testicular gene expressions in Hu sheep in summer, a total of 144 male Hu sheep aged four months were randomly divided into three groups (Con, Cur1, and Cur2, n = 48). Sheep in Con, Cur1, and Cur2 groups were fed a basal diet supplement with 0, 450, and 900 mg (per sheep) curcumin daily, respectively. Sheep were fed for 35 days, including a pre-feed for seven days. The results showed that the supplement with 450 mg and 900 mg curcumin increased serum free fatty acid (NEFA) and glutathione peroxidase (GPX), as well as IgA and IgM. The supplement with 450 mg curcumin increased the IgG level, while the supplement with 900 mg curcumin had a lower IgG level than the supplement with 450 mg curcumin (*p* < 0.05). Dietary curcumin supplement increased testicular organ index, serum testosterone level, and testicular star mRNA expression (*p* < 0.05). Furthermore, dietary curcumin supplement linearly inhibited testicular apoptosis with increased testicular bcl-2 mRNA expression and decreased caspase-3 mRNA expression (*p* < 0.05). In conclusion, dietary curcumin supplement can promote lipid metabolism, antioxidant capacity, and immune response, as well as testicular development, in Hu sheep, which provides evidence of application of curcumin in sheep production.

## 1. Introduction

In summer in southern China, heat stress induced by high ambient temperature and humidity usually harms the growth and reproductive performance of animals. For male animals, the main manifestations are poor appetite, decreased feed intake and daily gain, lower sexual desire, decreased semen volume and sperm motility [1,2]. Usually, there will be some changes in the metabolism to prevent animals from greater harm by high ambient temperature [3,4,5]. When animals suffer from heat stress, their immune capacity will also decrease [6,7,8,9]. Numerous studies showed that heat stress induced oxidative stress, as reviewed by Nisar [10], which can cause DNA damage, cell apoptosis, and inflammation [11,12,13]. Other studies showed that heat stress can cause metabolic, immune, and reproductive disorders through the sympathetic-adrenal medullary system and the hypothalamic-pituitary-adrenal axis [14,15,16].

Recently, plant-derived antioxidants such as tomato pomace, betaine, turmeric rhizome powder, and Artemisia annua L were widely used as feed additives to enhance body function and alleviate stress damage [17,18,19,20]. Curcumin or curcuminoid of turmeric, a plant-derived substance, with the effect of scavenging oxidative free radicals due to its phenolic structure [21], can be used as an antioxidant in a daily dietary supplement. Studies have shown that curcumin has various effects, such as anti-cancer, anti-inflammatory, and anti-cardiovascular disease [21,22,23,24,25]. Curcumin also has a protective effect on DNA damage repair, prevention of spermatogenic cell apoptosis, and development of testicular tissue [26]. In the livestock, curcumin has been used to improve the enzyme activity, performance, egg quality, and anti-oxidative state [27,28,29]. It has been reported that nursing lambs fed diets with curcumin can significantly improve daily weight gain, production performance, metabolism, and immune performance [30].

Hu sheep is a local sheep breed in China with high reproductive characters [31]. However, in summer, the high temperature affects its growth and fertility. Previous studies have found that dietary betaine may have beneficial effects on sheep exposed to heat, and chestnut tannins can improve the meat quality, welfare, and antioxidant status of heat-stressed lambs [32,33]. Therefore, in this study, the effects of curcumin on the blood biochemical indexes and the expression of reproductive related genes in testis were evaluated during summer in Hu sheep, which provides evidence for the protective role of curcumin against heat stress in Hu sheep.

## 2. Materal and Methods

The investigational procedures involving Hu sheep were directed in agreement with the guide for the care and use of domestic animals organized by the Institutional Animal Care and Use Committee of Nanjing Agricultural University, China.

### 2.1. Animals and Chemicals

One hundred and forty male Hu sheep aged four months with bodyweight about 25.82 ± 0.34 kg were used in this study. Hu sheep were housed at temperature (33.32 ± 0.33°C) and humidity level (70.56% ± 1.26%). Curcumin with the purity of 98% was purchased from Zhengzhou Baisite Food Additive Co., Ltd (Zhengzhou, China).

### 2.2. Experimental Design

Hu sheep were randomly divided into three groups (n = 48), with three replicates in each group and 16 sheep per replicate. Sheep were fed basal diets supplemented with 0 mg (Con), 450 mg (Cur1), 900 mg (Cur2) curcumin (per sheep), respectively. The curcumin is mixed with the concentrate, the concentrate is then fed first and after that, the roughage is fed. Basal diets were formulated in Table 1. After the test with pre-feed for one week and formal feed for four weeks, sheep were weighed. The blood samples were collected to make serum and plasma, and the testes were collected and weighed, and then pieces were stored at −80℃ for further gene expression analysis.

#### 2.2.1. Body and Testis Weight

At the end of the experiment, four sheep were randomly selected from each replicate to measure their body weight (n = 12) and the testes were castrated to measure their weight (n = 4).

#### 2.2.2. Serum Biochemical Parameters

The serum concentration of glucose (Glu), non-esterified acid (NEFA), triglyceride (TG), low-density lipoprotein (LDL), high-density lipoprotein (HDL), total cholesterol (TC) was determined using the corresponding kits (Nanjing Jiancheng Institute of Bioengineering Ltd., Nanjing, China).

#### 2.2.3. Serum Activity of SOD and GPX

The activity of the serum superoxide dismutase (SOD) and glutathione peroxidase (GPX) was determined using the corresponding kits (Nanjing Jiancheng Institute of Bioengineering Ltd., Nanjing China).

#### 2.2.4. The Plasma Concentration of IgA, IgM, and IgG

The plasma concentration of immunoglobulin A (IgA), immunoglobulin M (IgM) and immunoglobulin G (IgG) was determined using enzyme-linked immunosorbent assay method (ELISA, Nanjing Jiancheng Institute of Bioengineering Ltd., Nanjing China), respectively.

#### 2.2.5. Quantitative Real-Time PCR

The total RNA was extracted from frozen testes using Trizol Reagent (TaKaRa Biotechnology Co. Ltd., Dalian, China). The concentration and purity of RNA were determined with a spectrophotometer (NanoDrop 2,000c, Thermo Scientific, USA). For each sample, 5μg of total RNA was treated with DNase I (TaKaRa) to remove DNA, and then the cDNA was synthesized by reverse transcription on Veriti 9902 Thermal Cycler (Applied Biosystems, USA) using the PrimeScript RT Reagent kit (TaKaRa). The reactions were incubated for 15 min at 37 °C, followed by 5 s at 85 °C. Real-time PCR was performed using the QuantStudio^®^ 5 Real-Time PCR Design and Analysis System (Applied Biosystems, USA) with a SYBR® Premix Ex Taq™ Kit (TaKaRa Biotechnology Co. Ltd., Dalian, China). The reactions include TB Green Premix Ex Taq (10 μL), PCR Primers (0.8 μL), ROX Reference Dye II (0.4 μL), DNA template (2 μL) and sterilized water with a final volume of 20 μL, and the cycling conditions were as follows: 95 °C for 30 s (Hold stage), followed by 40 cycles of 95 °C for 5 s and 60 °C for 30 s (PCR stage), then 95 °C for 15 s, 60 °C for one min, and 95 °C for 15 s (Melt curve stage). The primer sequence for the genes was obtained from the NCBI database (Table 2). The values of all genes’ mRNA expression were normalized to *actb* and were calculated using the 2 ^−ΔCT^ method.

#### 2.2.6. Plasma Testosterone Concentration

Plasma testosterone concentration was determined using ELISA kit (Nanjing Jiancheng Institute of Bioengineering Ltd., Nanjing China). The assay sensitivity was 0.02 ng/mL. The intra-assay and inter-assay coefficients of variation were < 10% and <15%, respectively.

#### 2.2.7. Statistical Analysis

All data are expressed as the mean ± standard error of the mean (SEM), and differences in means were analyzed by one-way analysis of variance (ANOVA) followed by Tukey’s multiple comparison test using SPSS version 22.0, and *p* < 0.05 means significant difference.

## 3. Result

### 3.1. Serum Biochemical Parameters

Curcumin supplement increased the serum concentration of NEFA, which was significantly higher in Cur2 than those in other groups (*p* < 0.05). However, curcumin supplement did not change serum concentrations of Glu, TG, LDL, HDL, and TC (Table 3).

### 3.2. Serum SOD and GPX activity

**Figure 1:**
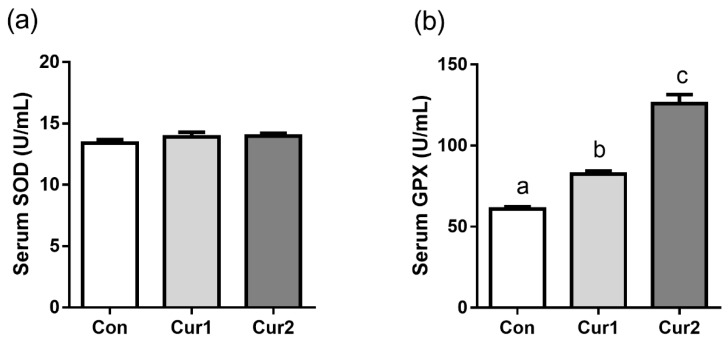
Effect of dietary curcumin supplement on the anti-oxidative capacity in Hu sheep in summer. Sheep in Con, Cur1, and Cur2 groups were fed basal diets supplemented with 0 mg, 450 mg, and 900 mg curcumin (per sheep) daily for 35 days, respectively. Serum was then collected to measure SOD (**a**) and GPX activity (**b**) by commercial kits. Data are shown as means ± SEM (n = 12). Bars with different letters denote significant differences (*p* < 0.05). SOD, superoxide dismutase; GPX, glutathione peroxidase.

Curcumin supplement increased serum activity of GPX, which was significantly higher in Cur2 than that in Cur1 (Figure 1a, *p* < 0.05). However, the activity of SOD was not significantly different in all groups (Figure 1b, *p* > 0.05).

### 3.3. Plasma Concentration of IgA, IgM, and IgG

**Figure 2:**
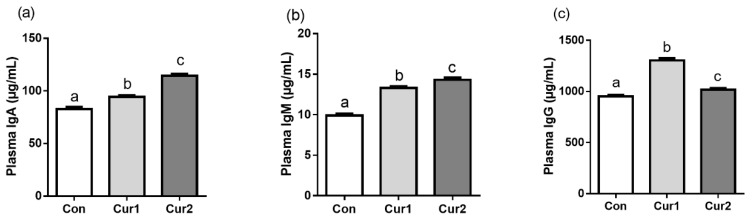
Effect of dietary curcumin supplement on the immune capacity in Hu sheep in summer. Sheep in Con, Cur1, and Cur2 groups were fed basal diets supplemented with 0 mg, 450 mg, and 900 mg curcumin (per sheep) daily for 35 days, respectively. Plasma was then collected to measure IgA (**a**), IgM (**b**) and IgG (**c**) concentration by ELISA. Data are shown as means ± SEM (n = 12). Bars with different letters denote significant differences (*p* < 0.05). IgA, immunoglobulin A; IgM, immunoglobulin M; IgG, immunoglobulin G.

Curcumin supplement increased the plasma level of IgA and IgM in a dose-dependent manner (Figure 2a,b, *p* < 0.05). Curcumin supplement also increased the plasma level of IgG. However, the plasma concentration of IgG in Cur1 was higher than that in Cur2 (Figure 2c, *p* < 0.05).

### 3.4. Testis Weight/Body Weight, Testosterone Concentration and Androgen-Related Gene Expressions

**Figure 3:**
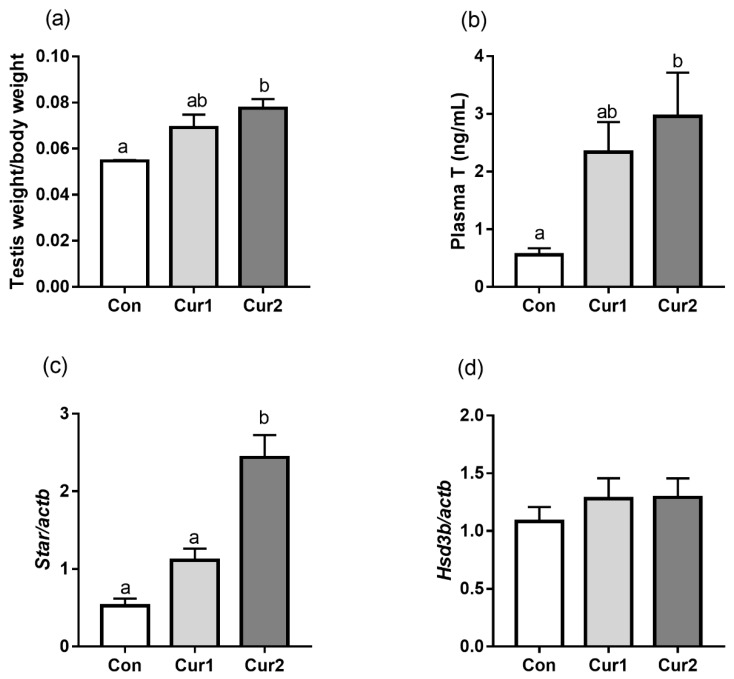
Effect of dietary curcumin supplement on testosterone concentration and testicular steroidogenic gene expressions in Hu sheep in summer. Sheep in Con, Cur1, and Cur2 groups were fed basal diets supplemented with 0 mg, 450 mg, and 900 mg curcumin (per sheep) daily for 35 days, respectively. Testes weight normalized to body weight (**a**). Plasma testosterone concentration (**b**) was detected by ELISA (n = 12). Testicular star mRNA (**c**) and hsd3b mRNA (**d**) expressions were analyzed by quantitative real-time PCR (n = 4), the y-axis scale is folded relative to actb. Data are shown as means ± SEM. Bars with different letters denote significant differences (*p* < 0.05). T, testosterone; star, steroidogenic acute regulatory protein; hsd3b, 3β-hydroxysteroid dehydrogenase; actb, β-Actin.

Curcumin supplement increased the relative testis weight (TW/BW) in Cur2 compared with those in Con and Cur1 (Figure 3a, *p* < 0.05), while there was no significant difference in TW/BW between Con and Cur1 (*p* > 0.05). Similarly, testosterone in plasma also had significant differences in Con and Cur2 (Figure 3b). The testicular star mRNA level in Cur2 was significantly higher than those in Cur1 and Con (Figure 3c, *p* < 0.05). However, there was no significant difference in the testicular hsd3b mRNA expression among all the groups (Figure 3d, *p* > 0.05).

### 3.5. Expression Patterns of Apoptosis-Related Genes in Testis 

**Figure 4:**
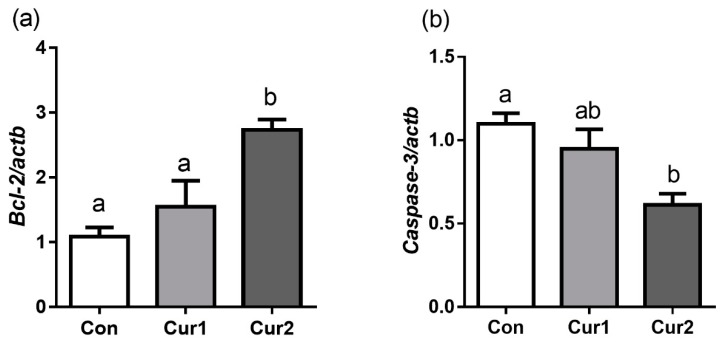
Effect of dietary curcumin supplement on testicular apoptotic gene expressions in Hu sheep in summer. Sheep in Con, Cur1, and Cur2 groups were fed basal diets supplemented with 0 mg, 450 mg, and 900 mg curcumin (per sheep) daily for 35 days, respectively. Testicular tissues were collected to detect anti-apoptotic bcl-2 (**a**) and apoptotic caspase-3 (**b**) mRNA expressions by quantitative real-time PCR. Data are shown as means ± SEM (n = 4). Bars with different letters denote significant differences (*p* < 0.05). The y-axis scale is folded relative to actb. bcl-2, B-cell lymphoma-2; actb, β-Actin.

Testicular bcl-2 mRNA expression in Cur2 increased compared with Con and Cur1 (*p* < 0.05). However, there was no significant difference between Con and Cur1 (Figure 4a, *p*>0.05). Conversely, the testicular expression level of caspase -3 gene in Cur2 decreased significantly compared with Con and Cur1 (*p* < 0.05), and there was no significant difference between Con and Cur1 (Figure 4b, *p* > 0.05).

## 4. Discussion

A previous study in nursing lambs showed that diets supplemented with curcumin can improve the growth performance, metabolism, and immune response. The current study investigates the effect of summer dietary curcumin supplement on the blood metabolites and testicular development in Hu sheep.

The biochemical parameters in serum can reflect animal metabolic status. A previous study has reported that heat stress can affect metabolism, which decreased the serum concentration of NEFA but not Glu in growing pigs [4]. Exposure to high heat load did not affect the concentrations of Glu, TG or NEFA in Australian Merino sheep [34]. However, curcumin supplement increased hepatic NEFA concentration and decreased plasma LDL cholesterol and hepatic TG concentrations in broiler chickens [35]. Curcumin also decreased the concentrations of plasma TG, TC, LDL cholesterol, and increased plasma HDL cholesterol and caused transcriptional inhibition of HMG-CoA reductase in mice fed high cholesterol [36]. Numerous studies, as reviewed by Zingg, showed curcumin can lower blood lipid levels [37]. It is reported that grape seed extract decreased serum TC, TG, LDL, and VLDL concentration, and vitamin C supplement decreased serum TC concentration in broilers suffering from heat stress [38]. The current increase in serum concentration of NEFA indicates that dietary curcumin supplement can promote fat mobilization in Hu sheep. Plasma cholesterol is transported back to the liver for metabolism by HDL, while it is transported to tissues by LDL. It is generally believed that higher HDL and lower LDL are beneficial to the organism. However, dietary curcumin supplement in the current study had no effect on Glu, TC, TG, HDL, and LDL, which might be due to the animal species and the dosage of curcumin. A study showed that different dosages of curcumin have different effects on serum biochemistry of nursing lambs and curcumin exerted few effects on hematological parameters [39] in which only 200 mg/kg curcumin increased serum levels of glucose and urea on day 30 of experiment, while both 100mg/kg and 200 mg/kg decreased serum levels of total protein and globulin and increased the cholesterol levels on days 15 and 30.

Heat stress usually induces ROS (reactive oxygen species), which can lead to oxidative stress [40]. SOD and GPX are antioxidant parameters that can scavenge ROS, and their activities are usually used to evaluate antioxidant ability in testis [41]. A previous study has reported that curcumin can up-regulate the relevant antioxidant enzymes through the Nrf-2 gene to alleviate the damage caused by heat stress, and increase the activity of SOD and GPX in mouse testis [42]. Studies have shown curcumin supplement can increase the serum SOD and GPX in poultry and sheep [43,44]. It is reported that low-dose curcumin-loaded Eudragit L-100-nanocapsules in the diet of dairy sheep increases antioxidant levels and reduces lipid peroxidation in milk [39]. Other studies showed the levels of MDA, and activities of lactate dehydrogenase and nitric oxide synthase were reduced, and the levels of T-AOC and GSH were enhanced when mouse fed the Mung bean flavonoids. while dietary resveratrol increased the activities of GSH-PX, SOD, CAT, and decreased the MDA level in serum of black-boned chickens and rats [45,46]. The current increase in GPX activity indicates that dietary curcumin supplement can improve the antioxidant capacity though it did not affect the serum activity of SOD in Hu sheep, and curcumin enhances antioxidant status by increasing GPX not SOD in Hu sheep.

Numerous studies, as reviewed by Aggarwal, have shown that heat stress decreased immune performance in animals [47]. Heat stress can change the body metabolic status accompanied by inflammation to weaken the immune function [48]. Studies have shown that heat stress can reduce the concentration of IgA, IgM, and IgG in poultry [49,50]. Other studies found that diets supplemented with tomato pomace, betaine, and turmeric rhizome powder increased the concentrations of total antibody, IgM, and IgG in the serum of broilers exposed to heat stress [17,19]. Dietary curcumin supplement can increase the serum concentration of IgG in lactating cows reared under high ambient temperature environment [51]. The current result of the increase in serum concentration of IgA, IgG, and IgM after curcumin supplement in Hu sheep was consistent with the previous study that curcumin increased IgG and IgM levels in rats under 2,3,7,8 Tetrachlorodibenzo -p-dioxin (TCDD) exposure [52]. However, the decreased IgG level in a high dosage of curcumin group might be a potentially toxic effect of curcumin [53,54] which needs further study.

Animals in high ambient temperature environment usually have poor testicular development compared with that in a normal environment. A previous study has shown that heat stress causes a reduction in testicular weight and desquamation of germ cells from seminiferous tubules, and the diameter of the seminiferous tubules becomes smaller, spermatogenic cells are shed, and the spermatogenic cells are arranged irregularly [55]. Trans-Resveratrol can increase sperm output, testosterone, and promote testicular development in healthy rats [56]. It is reported that curcumin dose-dependently improved spermatogenic disorders induced by scrotal heat stress in mice, and vitamin E promoted the development of reproductive organs in Boer goat [55,57]. Therefore, the current increase in relative testicular weight after dietary curcumin supplement might indicate that curcumin can stimulate testicular development in summer.

Testosterone is synthesized and secreted by interstitial Leydig cells in testes [58]. The Leydig cells synthesize steroid hormones from cholesterol which is the same as all other steroid-producing cells [59]. Testosterone synthesis is regulated by relative steroid-producing genes such as star and hsd3b [60]. Previous studies showed heat stress could decrease the star mRNA expression in mice testes [61] and dietary supplemented with curcumin can increase testicular hsd3b mRNA expression and the concentration of testosterone in heat stress in mice [62]. Studies also showed flavonols and quercetin derivative compounds increased star gene expression in MA-10 Leydig cells [63,64]. The current increase in serum testosterone concentration and testicular star mRNA expression might indicate that dietary curcumin supplement can improve the reproductive performance of Hu sheep.

In hot summer, the high temperature of the scrotum leads to testicular cell apoptosis, spermatogenic disorders, and male infertility [65,66]. Previous studies have shown that heat stress can reduce testicular bcl-2 mRNA expression in pigs [67], and increase caspase-3 gene expression in rats [42], and dietary lycium polysaccharide supplement increased testicular bcl-2 mRNA expression and decreased caspase-3 mRNA gene expression after heat stress in rats [68], which was consistent with the study of Lin C that curcumin has the effect on anti-apoptosis [55]. Curcumin can alleviate testicular damage, cell apoptosis, and spermatogenic cell death induced by streptozotocin and aflatoxin [69,70]. Therefore, the current increase in bcl-2 and decrease in caspase-3 gene expression indicate that dietary curcumin supplement can alleviate the testicular cell apoptosis in sheep production in summer.

## 5. Conclusions

Dietary curcumin supplement with 450 and 900 mg (per sheep) can increase NEFA and GPX in serum, which indicates curcumin promotes lipid metabolism and improves antioxidant status in Hu sheep. Dietary curcumin supplement improves immune ability by increasing the concentrations of IgA, IgM, and IgG in plasma. Results of the increase of testosterone in plasma, star, and hsd3b genes expression showed curcumin dose-dependently improves the reproductive performance of Hu sheep. The current increase in bcl-2 and decrease in caspase-3 gene expression showe that curcumin can prevent testicular cell apoptosis.

## Figures and Tables

**Table 1:** Formula and nutritional composition of the basal diet (based on dried matter).

Item	Content/%	Item	Content
Concentrate			
Corn	51.22	DE (MJ/Kg)	12.81
Wheat bran	17.41	CP (g/Kg)	171.73
Barley bran	15.91	Ca/%	0.85
Sesame	4.98	P/%	0.66
Premix	6.46		
NaHCO_3_	2.48		
Salt	1.49		
NaSeO_3_	0.05		
Total	100		
Roughage			
Silage	52.24	DE (MJ/Kg)	3.79
Hay	29.85	CP (g/Kg)	49
Soybean milk residue	17.91	Ca/%	0.4
Total	100	P/%	0.07

Note: Ingredients contained in each kilogram of premix: Vitamin A 80 KIU/kg, vitamin D_3_ 20 KIU/kg, vitamin E 200 mg/kg, Fe 640 mg/kg, Mn 640 mg/kg, Cu 120 mg/kg, Zn 640 mg/kg, Co 2.5 mg/kg, I 10.5mg/kg, Se 2.5mg/kg. DE, digestible energy; CP, crude protein; Ca, calcium; P, phosphorus.

**Table 2:** Primers used for quantitative real-time PCR analysis of genes expressions.

Gene	Primer Sequence (5′-3′)	Product Size (bp)	GeneBank Accession Number
*star*	F: GGGCATCCTCAAAGACCAG	120	NM_001009243.1
	R: TCCACCACCACCTCCAAC		
*hsd3b*	F: ATCCACACCAGCACCATAG	144	NM_001135932.1
	R: TTCCAGCACAGCCTTCTC		
*bcl-2*	F: CGCATCGTGGCCTTCTTT	113	XM_027960877.1
	R: CGGTTCAGGTACTCGGTCATC		
*caspase-3*	F: TCAGGGAAACCTTCACGAGC	274	XM_027962551.1
	R: CCTCGGCAGGCCTGAATAAT		
*actb*	F: CCAAGGCCAACCGTGAGAAG	349	NM_001009784.3
	R: CCATCTCCTGCTTCGAAGTCC		

Note: star, steroidogenic acute regulatory protein; hsd3b, 3β-hydroxysteroid dehydrogenase; bcl-2, B-cell lymphoma-2; actb, β-Actin.

**Table 3:** Effect of curcumin supplement on serum biochemical indices in Hu sheep (means ± SEM, n = 12).

Item	Con	Cur1	Cur2
Glu (mmol/L)	3.37 + 0.81	3.30 + 0.89	3.81 + 1.17
TC (mmol/L)	3.00 + 0.43	3.26 + 0.36	3.30 + 0.52
TG (mmol/L)	0.31 + 0.08	0.33 + 0.06	0.34 + 0.08
NEFA (mmol/L)	0.26 + 0.07 ^a^	0.30 + 0.08 ^ab^	0.36 + 0.09 ^b^
HDL (mmol/L)	0.85 + 0.14	0.88+0.11	0.87 + 0.10
LDL (mmol/L)	0.71 + 0.11	0.73 + 0.15	0.71 + 0.16

Note: ^a,b^ Values with different superscript means significant difference (*p* < 0.05).Glu, glucose; TC, total cholesterol; TG, triglyceride; NEFA, non-esterified acid; HDL, high-density lipoprotein; LDL, low-density lipoprotein.

## Date Availability Statement

The data that support the findings of this study are available from the corresponding author upon reasonable request.
